# Dated Phylogeny of *Astragalus* Section *Stereothrix* (Fabaceae) and Allied Taxa in the Hypoglottis Clade

**DOI:** 10.3390/biology12010138

**Published:** 2023-01-16

**Authors:** Ali Bagheri, Ali Asghar Maassoumi, Jonathan Brassac, Frank R. Blattner

**Affiliations:** 1 Department of Plant and Animal Biology, Faculty of Biological Science and Technology, University of Isfahan, Hezarjarib Street, Isfahan P.O. Box 81746-73441, Iran; 2 Botany Research Division, Research Institute of Forests and Rangelands, Agricultural Research, Education and Extension Organization (AREEO), Tehran P.O. Box 13185-116, Iran; 3 Leibniz Institute of Plant Genetics and Crop Plant Research (IPK), D-06466 Gatersleben, Germany; 4 Institute of Agricultural and Nutritional Sciences, Martin Luther University, D-06120 Halle, Germany; 5 Julius Kühn Institute (JKI), D-06484 Quedlinburg, Germany; 6 German Centre of Integrative Biodiversity Research (iDiv) Halle-Jena-Leipzig, D-04103 Leipzig, Germany

**Keywords:** *Astragalus* subgenus *Hypoglottis*, Leguminosae, Iran, phylogeny, rapid diversification, section *Hypoglottidei*, section *Stereothrix*

## Abstract

**Simple Summary:**

Biological taxonomic research deals with the grouping of organisms into entities that reflect their evolutionary history and relationships. In the species-rich plant genus *Astragalus*, the systematic grouping of many species changed several times during recent decades, which indicates problems in correctly recognizing relationships based on morphological characteristics. Here, we analyzed the relationships of *Astragalus* species from Iran and neighboring countries based on DNA sequences from three different loci. We found that species traditionally classified into two different sections of *Astragalus* occur intermingled in our phylogenetic trees instead of forming clear groups reflecting their taxonomic units. In addition, species thought to be only distantly related to the target species were found in this cluster. From this, we conclude that the currently used circumscription of taxonomic entities for these *Astragalus* species is false and should be abandoned. The reasons behind the the systematic classification problems of *Astragalus* include independent, parallel evolution or the loss of characteristics that were assumed to be unique and used to define certain systematic units. Thus, it is necessary to analyze the relationships of many *Astragalus* species to (i) identify traits useful for taxonomic classification and (ii) to understand the ecological and habitat differences driving their fast speciation.

**Abstract:**

The *Astragalus* subgenus *Hypoglottis* Bunge, which consists of several sections, is one of the taxonomically most complicated groups in the genus. The *Astragalus* section *Stereothrix* Bunge belongs to this subgenus and is a significant element of the Irano-Turanian floristic region. A molecular phylogenetic analysis of this section and its closely related taxa using nuclear ribosomal DNA internal transcribed spacers (ITS) and external transcribed spacer (ETS) regions as well as plastid *mat*K sequences were conducted. Parsimony analyses and Bayesian phylogenetic inference revealed that the section is not monophyletic in its current form, as some taxa belonging to closely related sections such as *Hypoglottidei* DC. and the *Malacothrix* Bunge group within the sect. *Stereothrix* render it paraphyletic. Moreover, species groups belonging to sect. *Stereothrix* are placed in different clades within the phylogenetic tree of subgenus *Hypoglottis*, which indicates polyphyly, i.e., multiple independent origins of taxa placed in the sect. *Stereothrix*. Molecular dating of the group estimated an age of 3.62 (1.73–5.62) My for this assemblage with the major diversification events happening during the last 2 My. Many species groups separated only within the last 0.5 to 1 My. Based on morphological and molecular data, we discuss the phylogenetic relationships of the groups and synonymy of species. In addition, the included taxa of sect. *Hypoglottidei* are not monophyletic and include species belonging to sects. *Hololeuce*, *Koelziana*, *Malacothrix*, *Onobrychoideae,* and *Ornithodpodium* group within the sect. *Stereothrix* taxa. We conclude that only an analysis including all groups and nearly all species of the sections within the Hypoglottis clade can finally result in an new evolutionary-based system for these taxa.

## 1. Introduction

*Astragalus* L., harboring about 3000 species, is the largest genus among flowering plants [1,2]. The first comprehensive infrageneric classification of the genus *Astragalus* was done by Bunge, who described nine subgenus eras. Each subgenus, according to its habit and morphological characteristics, was subdivided into several sections [3,4]. Molecular investigations in the last two decades resolved a significant number of taxonomic problems at the sectional level [5,6,7,8,9]. However, the circumscription of some sections remains unresolved. In this study, we focus on taxa within the subgenus *Hypoglottis* Bunge, consisting of several sections. However, the taxonomic delimitation of sections and placement of many species are debated among researchers. The subgenus *Hypoglottis* contains woody and perennial herbaceous plants. The main taxa within this subgenus are sections *Hypoglottoidei* DC., *Malacothrix* Bunge, and *Stereothrix* Bunge, the latter being one of the most diverse sections within this subgenus. The major diagnostic characteristics of the section are the herbaceous caulescent growth form (rarely acaulescent), possession of basifixed hairs, imparipinnate leaves, stipules which are free from the petiole or shortly adnate to it, a non-inflated calyx, and rounded or emarginate wing blades [1]. According to the circumscription of Maassoumi [10,11,12], sect. *Stereothrix* is one of the medium-sized sections of *Astragalus*, with a total of 28 species.

Section *Stereothrix* has been taxonomically revised several times [1,10,11,12,13,14,15,16] but the taxonomic positions of some of the species suggested to belong to this section are unclear. In their account for Flora Iranica, Podlech et al. [13,14] treated sect. *Stereothrix* based on the diagnostic traits mentioned above. However, in a more recent revision of the genus, Podlech and Zarre [1] and Maassoumi [11,12,17] transferred a number of species from sect. *Stereothrix* to other closely related sections or vice versa (Table 1 and Table 2).

Based on the recent comprehensive molecular studies on the entire genus by Azani et al. [6] and Su et al. [18] nine and ten clades have been inferred, respectively. One important clade in both studies is the Hypoglottis clade which includes annual and perennial species [6]. There is some evidence in these phylogenies that several sections of *Stereothrix* and *Hypoglottoidei* are non-monophyletic, as shown by Azani et al. [5,6], but more work is evidently needed. Here, rarely studied taxa belonging to different sections of the subgenus *Hypoglottis* (according to Bunge) and/or the Hypoglottis clade (according to Azani et al. [6] and Su et al. [18]), with a focus on representative species of sect. *Stereothrix,* were selected for molecular analysis to solve taxonomic problems and arrive at better insights into their systematic positions. For this, DNA sequences of the internal and external nuclear ribosomal DNA (nrDNA) spacers (i.e., the ITS and ETS regions) and the plastid *mat*K gene were used as molecular markers.

## 2. Materials and Methods

### 2.1. Taxon Sampling

Herbarium dried leaf materials for DNA extraction from the *Astragalus* sect. *Stereothrix* and closely related taxa comprising most of the type specimens (16 species means about 45% of the total species sequenced here) were obtained from the relevant collections of the herbaria MSB, TARI, and W (herbarium acronyms follow Thiers [19]). In total, we included 83 individuals representing 60 species comprising 22 sects. *Stereothrix* and 29 species of the other related sections, plus 6 species from taxonomically distant taxa, including *A. annularis* (sect. *Annulares*), *A. echinops* and *A. alopecias* (sect. *Alopecuroidei*), *A. hymenostegis* (sect. *Hymenostegis*), *A. glaucacanthus* (sect. *Poterion*), and *A. compactus* (sect. *Rhacophorus*). Species from the genus *Oxytropis* DC. (*O. aucheri* and *O. pilosa*) as well as *Colutea persica* were included as outgroups. In addition, we obtained 26 sequences from GenBank for completing our datasets. Voucher specimen information and GenBank sequence accession numbers for the examined taxa are listed in Table S1 (Supplemental online information).

### 2.2. DNA Extraction, PCR Amplification, and DNA Sequencing

Total genomic DNA was extracted from dried herbarium leaf tissue with a DNeasy Plant DNA Extraction Kit (Qiagen) according to the instructions of the manufacturer. Lysis time was doubled in comparison to Qiagen’s protocol to account for the dry state of the herbarium-derived leaves. After DNA extraction, we checked DNA quality and concentration on 1.5% agarose gels. For the internal transcribed spacer (ITS) region, including ITS1, 5.8S rDNA, and ITS2, amplifications were done using primers ITS-A and ITS-B [20]. In addition, for old herbarium materials, the internal primers ITS-C and ITS-D, binding in the 5.8S rDNA [20], were used together with the before mentioned ITS primers for separate amplification of ITS1 and ITS2. For the 5′ external transcribed spacer (ETS) region upstream of the 18S rDNA, amplifications were done using primers ETS-cis2F and 18S-ETS [21]. Finally, for the chloroplast matK gene, amplifications were done using the primer pairs trnK685F/matK832R and matK4LaF/trnK2R* [22]. PCR amplification protocols for all markers by Bagheri et al. [7] were followed. Both nuclear regions and the plastid gene were directly Sanger sequenced on an ABI 3730 XL using the amplification primers.

### 2.3. Sequence Alignments

Forward and reverse sequences of ETS, ITS, and matK were assembled in Chromas v. 2.6.6 [23], manually corrected where necessary, and afterward aligned using Muscle version 3.8.425. Five datasets were generated, namely, each region separately, the concatenation of both nrDNA regions (ETS + ITS), and the three regions concatenated (ETS + ITS + matK).

### 2.4. Phylogenetic Inferences

Maximum parsimony (MP) analyses were conducted in Paup* 4.0a169 [24] using a two-step heuristic search, as described by Blattner [25] with 1000 initial random addition sequences (RAS). To test clade support, bootstrap analyses were run on all datasets with resampling 1000 times with the same settings as before, except that we did not use the initial RAS step. Paup* was also used to infer the best-fitting model of sequence evolution for the three marker regions (Table 3) using the Akaike information criterion (AICc).

Bayesian phylogenetic inference (BI) was conducted in MrBayes 3.2.6 [26] on the partitioned dataset specifying the respective models of sequence evolution for each data partition. In BI, two times four chains were run for 5 million generations for all datasets specifying the respective model of sequence evolution. In all analyses, we sampled a tree every 500 generations. Converging log-likelihoods, potential scale reduction factors for each parameter, and inspection of tabulated model parameters in MrBayes suggested that stationary had been reached in all analyses. The first 25% of trees of each run were discarded as burn-in.

### 2.5. Incongruent Length Difference Test

The congruence of the nrDNA and plastid datasets was evaluated using the partition homogeneity or incongruent length difference test (ILD) of Farris et al. [27] in Paup*. The test was run using the heuristic search option including the simple addition sequence and TBR branch swapping with 1000 homogeneity replicates with the elimination of invariant characteristics [28].

### 2.6. Divergence Time Estimation

The clade ages and divergence times among the investigated taxa were estimated using the crown-age for *Astragalus* of 14.36 million years (My). This age was obtained by Azani et al. [6] from a dating analysis based on ITS and chloroplast sequences from 110 representatives of *Astragalus* and papilionoid legumes from the Hologalegina clade. Azani et al. [6] used two calibration points inferred from Lavin et al.’s dating analysis of Leguminosae using 12 legume-specific fossils [29]. We used Beast 2.7.0 [30,31] to analyze the partitioned sequences. The site model and the phylogeny were co-estimated in a single Bayesian analysis as offered by the Beast package bModelTest [32]. This package not only reduces the number of steps to perform the phylogenetic analysis by integrating the model-testing phase in the main analysis but also incorporates the site model uncertainty into the phylogenetic posterior distribution. We used the uncorrelated log-normal relaxed clock, as provided by the Beast package optimized relaxed clock (ORC) [33,34], and the calibrated Yule prior [35,36]. Monophyly of the *Astragalus* clade was enforced and the node was defined with a normal-distributed prior (mean = 14.36, stdev = 2). Three independent analyses were run for 20 million generations each, sampling every 5000 generations. Tracer 1.7.2 was used to assess the convergence of the analyses and most parameters reached an effective sample size (ESS) of at least 200, indicating a good mixing of the Markov chain Monte Carlo (MCMC). The runs were combined with Logcombiner (part of the Beast software) discarding the initial 25% of each run as burn-in. A maximum clade credibility (MCC) tree was summarized with Treeanotator (part of the Beast software) using the option “Common Ancestor heights” for the nodes.

## 3. Results

### 3.1. Phylogenetic Analyses

**Figure 1:**
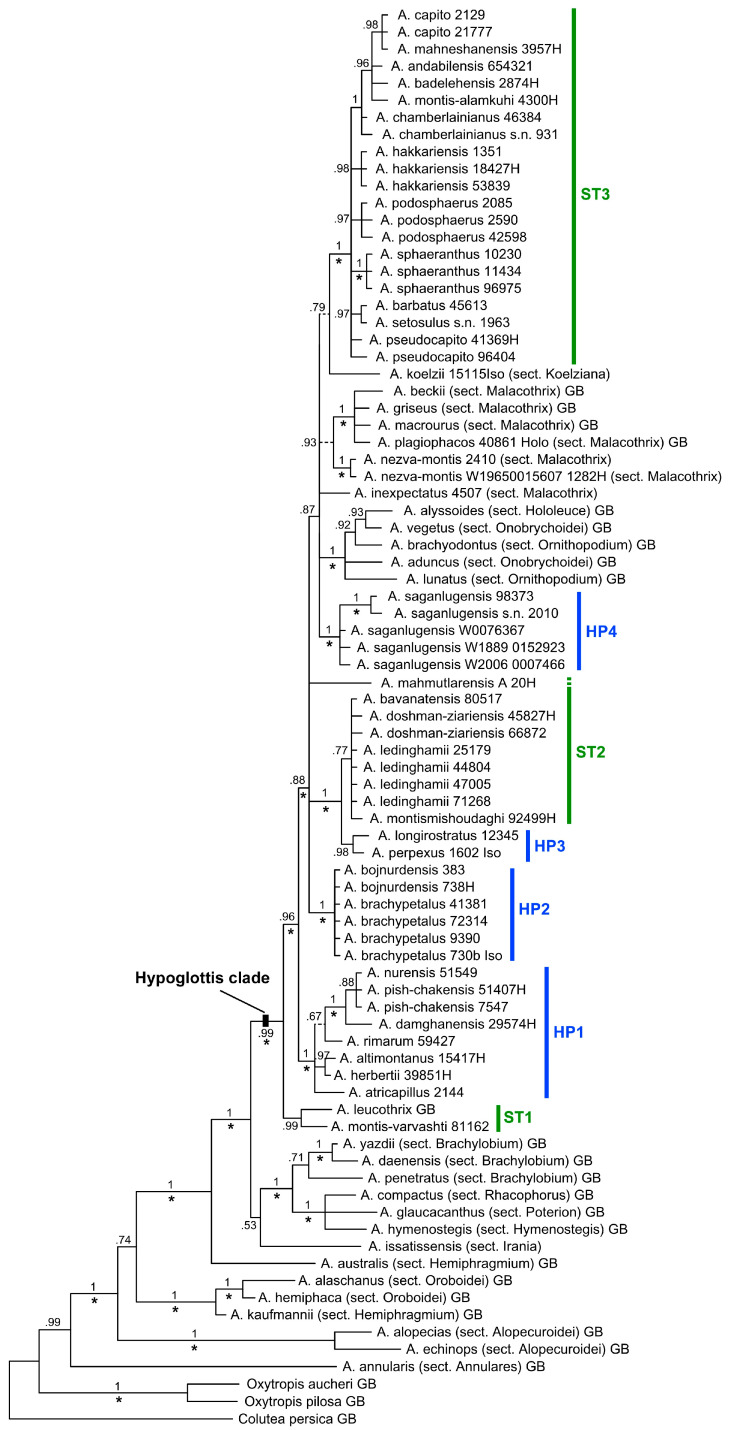
Bayesian phylogenetic tree based on the combined data matrix consisting of ETS, ITS, and *mat*K sequences. Numbers at branches indicate the Bayesian posterior probabilities. The tree topology is identical to the strict consensus tree of maximum parsimony (MP) analysis except for three clades (indicated by dashed branches) that were not recovered in MP. MP bootstrap support >70% is indicated by asterisks (*) at the respective branches. Sectional affiliation of species outside sects. *Stereothrix* (ST1–ST3) and *Hypoglottidei* (HP1–HP4) are given in brackets after the species’ names. GB indicates sequences that were obtained from the GenBank nucleotide database.

The aligned nrDNA ETS, ITS, combined dataset of ETS + ITS, matK, and combined dataset of ETS + ITS + matK matrices comprised 282, 618, 900, 1153, and 2053 bp across 83 accessions, respectively. The ILD test (*p* = 0.04) suggested no significant length incongruence between the nuclear and plastid markers, therefore, we also analyzed them as a combined dataset. The independent MP and BI analyses of the ITS + ETS and matK datasets produced consistent results differing only regarding the phylogenetic resolution of the obtained trees, which is higher in the nuclear dataset compared to matK. Hence, because of similar results of the analyses, only the total evidence Bayesian tree of the three combined marker regions along with its posterior probabilities (PP) and bootstrap values >70% [37] from MP analysis is shown here (Figure 1). Differences between the BI and MP analyses occurred at three positions in the tree, where BI resolved relationships with low support values that in the MP strict consensus tree resulted in polytomies. Characteristics of the analyzed datasets are provided in Table 3.

### 3.2. Phylogenetic Reconstructions and Age Estimates

Bayesian phylogenetic inference and Maximum Parsimony based on the combined dataset (ETS + ITS + *mat*K) resulted in a tree with several strongly to moderately supported subclades (Figure 1) within the large so-called Hypoglottis clade of Azani et al. [6] and Su et al. [18]. The two large sections within subgen. *Hypoglottis*, i.e., sects. *Hypoglottidei* (members marked as HP1–HP4 in Figure 1) and *Stereothrix* (ST1–ST3), are polyphyletic. Furthermore, taxa of sects. *Onobrychoidei*, *Ornithopodium*, and *Hololeuce* forming the subgen. *Cercidothrix* Bunge group inside the Hypoglottis clade. The former two sections are not monophyletic. Regarding the subgen. *Hypoglottis* sections, where we included multiple species, relationships of taxa are not completely resolved.

Non-monophyly was also detected outside the Hypoglottis clade where *A. australis* and *A. kaufmannii*, the two analyzed members of the sect. *Hemiphragmium,* are nested within two different clades.

In our BI tree, not all species belonging to taxonomically distant sections are resolved as sister taxa of the Hypoglottis clade. Species from sects. *Ornithopodium*, *Onobrychoidei,* and *Hololeuce* of subgen. *Cercidothrix* were found nested within this clade so that the boundaries of subgen. *Hypoglottis* in the current circumscription is also not clear. The remainder of the taxa belonging to subgen. *Hypoglottis* form a large polytomy, with members of the sect. *Stereothrix* mostly placed in three groups categorized as ST1–ST3 (Figure 1) and intermingled with taxa of other sections, mostly from sections *Hypoglottidei* and *Malacothrix*.

**Figure 2:**
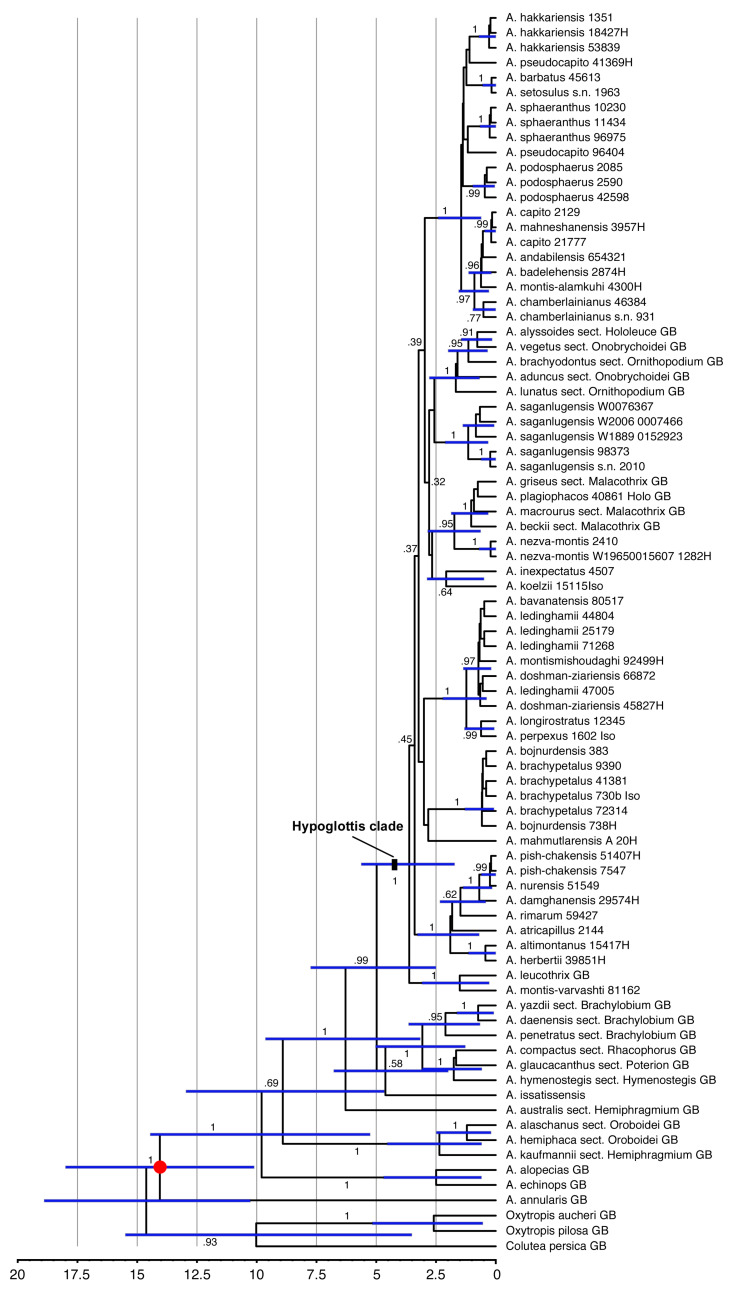
Dated phylogeny calculated from the combined ETS, ITS, and *mat*K sequences using the calibrated Yule prior and a secondary calibration on the crown group of *Astragalus* of 14.6 million years (red dot). Node bars indicate 95% highest probability density intervals (HPD) for the ages. The scale provides a timeline in million years before the present. Numbers along the branches give posterior probabilities.

Age estimations for the clades within our set of *Astragalus* taxa (Figure 2) arrived at a crown age of 3.62 (1.73–5.62) My for the Hypoglottis clade, with the major diversification events within sects. *Stereothrix* and *Hypoglottidei* occurring during the last 2 My and many species originating only during the last 500,000 years.

## 4. Discussion

### 4.1. Non-Monophyly of Bunge’s Traditional Subgenus Hypoglottis vs. Monophyly of the Hypoglottis Clade

Subgenus *Hypoglottis* was established by Bunge [3,4] as comprising sects. *Stereothrix*, *Hypoglottidei*, *Malacothrix,* and *Dasyphyllium*. Our results confirm that these sections are closely related and belong to the Hypoglottis clade, an informal unit that most closely resembles Bunge’s subgen. *Hypoglottis*. Ranjbar and Karamian [38] transferred two other sections (*Hemiphaca*, also treated as sect. *Oroboidei*, and *Hemiphragmium*) to subgen. *Hypoglottis*. However, our results show that these taxa clearly fall outside the Hypoglottis clade and that sect. *Hemiphragmium* is even not monophyletic (Figure 1). In contrast, species of sects. *Hololeuce*, *Onobrychoidei,* and *Ornithopodium*, all belong to the subgen. *Cercidothrix*, grouped within the Hypoglottis clade, rendering subgen. *Hypoglottis* sensu Bunge paraphyletic. For these outgroup taxa, we included only a few species so we cannot draw further conclusions here.

Maassoumi et al. [39], in their infrageneric system of *Astragalus*, classified all the above taxa in Clade VII, including taxa belonging to subgen. *Hypoglottis* and *Cercidothrix* together with some annual species from sects. *Sesamei*, *Hispiduli*, *Dipelta*, *Mirae*, *Platyglottis*, *Heterodontus*, *Ankylotus*, *Pentaglottis*, *Ophiocarpus,* and New World aneuploids (Neo-*Astragalus*) [39]. This placing is also reflected in other studies [5,6,18]. There is considerable previous evidence in other papers that subgenus *Cercidothrix* is not monophyletic [18], and that the type species is actually in a completely different clade (Hamosa clade). By considering these approaches, Clade VII in Maassoumi et al. [39] and the Hypoglottis clade can be assumed to be monophyletic, particularly as the latter receives high support values (PP 1, MP bootstrap support 100%) in our analysis.

### 4.2. Divergence Times and Fast and Young Diversification

The Hypoglottis clade is characterized by large polytomies. The lack of phylogenetic resolution within this part of our data (Figure 1) is interpreted as evidence for the rapid and simultaneous evolutionary radiation of the involved taxa about 3 (1.9–4) My ago in the Upper Pliocene, resulting in a so-called hard polytomy in the phylogenetic trees. While morphological differentiation within this clade is partly pronounced, the morphological radiation was not accompanied by a similar variation in the molecular marker regions we used for our study. A Middle Pliocene (~4 My ago) diversification in the Irano-Turanian steppe regions was suggested for sect. *Hymenostegis* [7]. Azani et al. [6] also reported the divergence of the main clades of *Astragalus* from the Middle Miocene to the Pleistocene. In their study, the divergence time estimate for the crown age of the Hypoglottis clade is 8.36 (6.29–10.54) My. However, here, we arrived at a younger age estimate for the clade harboring sects. *Stereothrix* and *Hypoglottidei* with a crown age of 3.62 (1.73–5.62) My. The discrepancy can be explained by the taxa included in the analyses. Azani et al. [6] considered basal species originating from Eastern Asia as representatives of the Hypoglottis clade, while here the focus was on the Irano-Turanian floristic elements. The extant species of these groups are mostly estimated to have originated during the last 0.5 to 1 My when the climate fluctuated repeatedly between Pleistocene glacial and interglacial periods, resulting in changes between cold and dry and warmer and more humid conditions in western Asia. Plant populations in the Hypoglottis clade migrating to cope with changing conditions might have contributed to geographic isolation- and vicariance-driven speciation.

### 4.3. Non-Monophyly of Sections Stereothrix, Hypoglottidei, and Their Allied Taxa

Our analysis shows that neither sects. *Stereothrix* nor *Hypoglottidei* are monophyletic in their current circumscription. The taxa belonging to sect. *Stereothrix* are placed in three distinct subclades (ST1–ST3) in the main tree (Figure 1), while the examined taxa for the sect. *Hypoglottidei* fall into four subclades (HP1–HP4).

Section *Stereothrix* subclade ST1, consisting of *A. montis-varvashti* and *A. leucothrix*, is the sister group to all other taxa in the Hypoglottis clade studied here; the first species is an endemic taxon of northern parts of Turkey, which grows at a 500–800 m elevation, while the latter grows in the northern parts of Iran on Varvasht Mountain at a 4000 m elevation as an alpine species. Based on morphology, the taxa clearly belong to sect. *Stereothrix*, but in our phylogenetic analyses they are remote from the core of the sect. *Stereothrix* (ST3) taxa and their relations with other members of this section remain unclear.

The second subclade (ST2) includes four species (*A. bavanatensis, A. doshman-ziariensis, A. ledinghamii,* and *A. montismishoudaghi*) belonging to this section. All taxa occur at low elevations between 700 and 2500 m in the southwestern parts of Iran. In contrast, most species are at the core of the sect. *Stereothrix* (ST3), adapted to high-elevation habitats in the central and southwestern parts of Iran. In addition to the distinct geographical distribution (mostly central and southwestern Iran instead of northern Iran), this subclade shares some morphological characteristics, including the calyx covered with white hairs mixed with few black hairs, inflorescences that are long and cylindrical and only rarely globose, plus vegetative parts that are mostly covered with very asymmetrical hairs. Species in this group are also not always monophyletic. For example, *Astragalus montismishoudaghi* from northwestern Iran groups in a polytomy with, among others, four individuals of *A. ledinghamii* from the southwestern parts of Iran (Figure 1: ST2). In addition to their different distribution areas, their morphology is also slightly but consistently different (connected stipule to petiole vs. free; obovate standard vs. rhomboid; plus some additional differences in quantitative characteristics such as having a shorter stem height, stipule length, calyx length, and calyx teeth in *A. montismishoudaghi*). We interpret this as characteristics of very young species (Figure 2) which just started to differentiate from their close relatives in the northwest of Iran.

The third subclade (ST3) of sect. *Stereothrix* (PP 0.99, BS 84%) contains the type species of the section (*A. barbatus*). Most taxa occur at relatively high elevations of the Alborz Mountains and in the north and northwest of Iran plus southeastern Turkey. Only two species, namely *A. sphaeranthus* and *A. podosphaerus*, occur in the alpine zone of the Zagros Mountains in western Iran (in contrast to ST2 members). In addition to their shared geographical distribution, the core clade of sect. *Stereothrix* (ST3) is defined by their dense, multifloral, and globose inflorescences, and having leaflets with densely tomentose and spreading hairs.

In this subgroup, *A. badelehensis* was previously assumed to be synonymous with *A. capito* [1], but they differ from each other in some morphological characteristics, including wings blades rounded at the apex (non-obliquely emarginate), peduncle up to 3.5 cm, covered with subappressed hairs (vs. peduncle 0.5−2 cm, covered with spreading hairs), calyx covered with spreading white hairs (non-spreading white hairs mixed with few shorter black hairs). Our phylogenetic tree also shows that they group in different clades; therefore, we recognize *A. badelehensis* as a distinct species.

Section *Hypoglottidei* is, with more than 50 species [1,12,40], one of the medium-sized groups within *Astragalus*. Similar to sect. *Stereothrix*, the taxa belonging to sect. *Hypoglottidei* do not form a monophyletic group in our study, although only 14 species of this section were included. The first subclade (HP1) includes seven species and forms the core of the sect. *Hypoglottidei*. These species are distributed in northern Iran in the Alborz Mountains. Two taxa (*A. altimontanus* and *A. damghanensis*) belonging to the sect. *Stereothrix* group here, indicating non-monophyly of both taxa.

According to Maassoumi [10], the specimen number “Wendelbo & Assadi” 29574, (MSB and TARI) was considered to belong to *A. haematinus*, which is now a synonym of *A. nurensis* (sect. *Hypoglottidei*) [1,12]. Podlech [41] described the new species *A. damghanensis* based on the above-mentioned specimen and put it in sect. *Stereothrix*. Here, we analyzed this specimen and showed that the systematic position of *A. damghanensis* is incorrect in sect. *Stereothrix* as it groups with sect. *Hypoglottidei* taxa. In general, the species belonging to sect. *Stereothrix* grow in alpine areas, while taxa of sect. *Hypoglottidei* grow at lower elevations (*A*. *damghanensis* grows at around 450 m). Moreover, morphologically, *A*. *damghanensis,* by having a tubular calyx with subulate teeth and distinctly incised wing apices, shares sect. *Hypoglottidei* characteristics. Taking into account the evidence of the habitat, distribution, and morphology of this species, together with its position in our molecular analysis, we can state that it is much closer to the traditional sect. *Hypoglottidei* species than to sect. *Stereothrix*.

The second subclade (HP2) consists of *A. brachypetalus* (with four individuals) and *A. bojnurdensis* (with two individuals). The first species is widely distributed in northeastern Iran and Turkmenistan while the latter is restricted to only a small area in northeastern Iran. Both species have important common morphological features (long calyx teeth and dense to lax globose inflorescences) which separate them from sect. *Hypoglottidei*. According to Podlech and Zarre [1], these taxa should either be placed in sect. *Stereothrix* or sect. *Brachylobium* based on their morphological characteristics. We assume them to be closer to sect. *Hypoglottidei* taxa (as in Maassoumi [11,12]), however, more studies are needed to determine the exact taxonomic and phylogenetic position of these species.

The third subclade (HP3) including *A. longirostratus* and *A. perpexus* (the latter one is synonymous) from sect. *Hypoglottidei* is placed here with high support values as a sister group to ST2, the species that were transferred by Podlech et al. [14] and Podlech and Zarre [1] from sect. *Hypoglottidei* to sect. *Oroboidei* and sect. *Hemiphaca*, respectively (see Table 1 and Table 2). This taxon grows in the Zagros Mountains in Lorestan, Chaharmahal and Bakhtiari, and Isfahan provinces of Iran. It is easily distinguishable from other members of sect. *Hypoglottidei* by having deeply incised and bicornuate wing petals. For us, the status of this species is not finally resolved and future studies on this taxon are needed.

The fourth subclade of sect. *Hypoglottidei* taxa (HP4) falls within a large polytomy together with clade ST3, plus species from diverse sections including sects. *Onobrychoidei*, *Hololeuce*, *Ornithopodium,* and *Malacothrix*. It is formed by *A. saganlugensis* with five individuals. This species occurs mostly in Turkey, Armenia, Azerbaijan, and a small area in northwestern Iran [42]. Foliaceous and green stipules are unique features of this species. Finally, the polytomy harboring HP4 and ST3 also contains species of sect. *Malacothrix* and sections belonging to subgen. *Cercidothrix* (sects. *Ornithopodium*, *Onobrychoidei,* and *Hololeuce*). Section *Malacothrix*, with more than 150 species [1,11], is one of the largest groups within *Astragalus*. In this study, we included just a few species in our dataset. Additionally, *A. nezva-montis* from sect. *Hypoglottidei* and *A. plagiophacos* from sect. *Plagiophaca* are nested in this subclade. More recently, Maassoumi [11] transferred two taxa (*A. nezva-montis* and *A. inexpectatus*) to section *Plagiophaca*, but here, we considered them as members of sect. *Malacothrix*. Our results support the notion that not only does the sectional division of *Astragalus* seem to be partly questionable but that some subgenera also might not reflect the evolutionary history of the taxa [6,18,21].

One remarkable species is *A. koelzii* that, in Figure 1, is sister to ST3 and, in Figure 2, is sister to *A. inexpectatus,* although in both cases with very low support. This species, by having unifoliolate leaves, is easily discernable from other members of sect. *Stereothrix*. It grows in an oak forest (*Quercus brantii*) in the Khuzestan province of Iran. Sirjaev and Rechinger [43] placed it in the monotypic sect. *Koelziana*, but Podlech et al. [14] and Podlech and Zarre [1] included this monotypic section as synonyms of sect. *Stereothrix*. Recently, Maassoumi [11] revived sect. *Koelziana* as a separate section within *Astragalus*. Here, in our molecular study, we included material taken from the type specimen of this taxon. Our efforts to find more individuals of this species in the vicinity of the type locality unfortunately failed. Maassoumi [12] transferred two other species (*A. doshman-ziariensis* and *A. ledinghamii*) from sect. *Stereothrix* to sect. *Koelziana*, a relationship that our results do not support. It is certain that *A. koelzii*, with its different morphological features, is closely related but distinct from other members of sect. *Stereothrix*, which supports a monotypic sect. *Koelziana*, but a definitive interpretation of the phylogenetic and taxonomic position of this taxon needs further study.

## 5. Conclusions

Our phylogenetic analysis focusing on rarely-studied species from the Irano-Turanian flora confirms that the infrageneric classification of *Astragalus* in sects. *Stereothrix* and *Hypoglottidei* is false. We also find clear evidence that non-monophyly is far-reaching regarding the sections and even subgenera within the Hypoglottic clade. This finding is in accord with earlier studies, resulting in similar groupings identified as non-monophyletic [5,6,18]. However, an increase in taxonomical sampling seems to have the highest priority to uncover the extent to which these groups are non-monophyletic and eventually define monophyletic units within this clade. Although we remark here on changes regarding the sectional affiliation of certain critical taxa, it is obvious that, due to repeated parallel evolution and/or loss of morphological traits and the young age of many species (mostly less than 1 My old), it is not possible to classify the examined taxa into the existing morphology-defined sections. What can be concluded is that the fast biological radiation resulting in high species numbers of *Astragalus* is ongoing in different geographical areas of western Asia, where diverse climatic conditions might contribute to speciation. However, this alone cannot be the main driver of diversification, as other plant groups co-occurring with the local *Astragalus* species do not show similar species richness in the study area. With regard to the intrageneric system for the analyzed taxa, we can only suggest abandoning the current system and merging all of the above-mentioned sections into a larger and monophyletic entity. To achieve the goal of a comprehensive circumscription not only in the Hypoglottis clade but probably also in many other *Astragalus* series from western to central Asia, the use of genome-wide DNA sequences seem necessary to increase the resolution within the phylogenetic trees and better discern hard polytomies from badly resolved tree parts due to a low number of available characteristics [6,44]. Only based on such a resolved dataset might we arrive at a better understanding of the reasons for the rapid speciation in *Astragalus* and the evolutionary trajectory of the morphological and ecological characteristics that might define infrageneric groups.

## Figures and Tables

**Table 1:** Taxonomic treatment history of taxa belonging to sect. *Stereothrix* and its allies.

Species	Podlech et al., 2010 [13]	Podlech et al., 2012 [14]	Podlech and Zarre 2013 [1]	Maassoumi 2018 [11]	Maassoumi 2020 [12]	Current Study
*A. altimontanus* Podlech and Maassoumi	*Stereothrix*	*Stereothrix*	*Stereothrix*	*Stereothrix*	*Stereothrix*	*Hypoglottidei*
*A. andabilensis* Ranjbar and Mahmoudian	*-*	*-*	*-*	-	*Stereothrix*	*Stereothrix*
*A. atricapillus* Bornm.	*Hypoglottidei*	*Hypoglottidei*	*Hypoglottidei*	*Hypoglottidei*	*Hypoglottidei*	*Hypoglottidei*
*A. badelehensis* Maassoumi and Taheri	*-*	*-*	*-*	*Stereothrix*	*Stereothrix*	*Stereothrix*
*A. barbatus* Lam.	*Stereothrix*	*-*	*Stereothrix*	-	*Stereothrix*	*Stereothrix*
*A. bavanatensis* Zarre and Podlech	*Stereothrix*	*Stereothrix*	*Stereothrix*	*Stereothrix*	*Stereothrix*	*Stereothrix*
*A. bojnurdensis* Podlech	*Brachylobium*	*Brachylobium*	*Brachylobium*	*Hypoglottidei*	*Hypoglottidei*	*Hypoglottidei*
*A. brachypetalus* Trautv.	*Hypoglottidei*	*Hypoglottidei*	*Stereothrix*	*Hypoglottidei*	*Hypoglottidei*	*Hypoglottidei*
*A. capito* Boiss. and Hohen.	*Stereothrix*	*Stereothrix*	*Stereothrix*	*Stereothrix*	*Stereothrix*	*Stereothrix*
*A. chamberlainianus* H.Sümbül	*-*	*-*	*Stereothrix*	-	-	*Stereothrix*
*A. daenensis* Boiss.	*Brachylobium*	*Brachylobium*	*Brachylobium*	*Brachylobium*	*Brachylobium*	*Brachylobium*
*A. damghanensis* Podlech	*Stereothrix*	*Stereothrix*	*Stereothrix*	*Hypoglottidei*	*Stereothrix*	*Hypoglottidei*
*A. doshman-ziariensis* Maassoumi and Podlech	*Stereothrix*	*Stereothrix*	*Stereothrix*	*Stereothrix*	*Koelziana*	*Stereothrix*
*A. hakkariensis* Podlech	*Stereothrix*	*-*	*-*	*Stereothrix*	*Stereothrix*	*Stereothrix*
*A. herbertii* Maassoumi	*Hypoglottidei*	*Hypoglottidei*	*Hypoglottidei*	*Hypoglottidei*	*Hypoglottidei*	*Hypoglottidei*
*A. inexpectatus* Maassoumi and Podlech	*Malacothrix*	*Malacothrix*	*Malacothrix*	*Malacothrix/ Plagiophaca*	*Plagiophaca*	*Malacothrix*
*A. koelzii* Barneby	*Stereothrix*	*Stereothrix*	*Stereothrix*	*Koelziana*	*Koelziana*	*Koelziana*
*A. ledinghamii* Barneby	*Stereothrix*	*Stereothrix*	*Stereothrix*	*Stereothrix*	*Koelziana*	*Stereothrix*
*A. leucothrix* Freyn and Bornm.	*-*	*-*	*Stereothrix*	-	*Stereothrix*	*Stereothrix*
*A. longirostratus* Pau (*=A. perpexus* Maassoumi)	*Hhypoglottidei*	*Hemiphaca*	*Oroboidei*	*Hypoglottidei*	*Hypoglottidei*	*Hypoglottidei*
*A. mahmutlarensis* Podlech	*-*	*-*	*Stereothrix*	-	*Stereothrix*	*Stereothrix*
*A. mahneshanensis* Maassoumi and Moussavi	*-*	*Stereothrix*	*Stereothrix*	*Stereothrix*	*Stereothrix*	*Stereothrix*
*A. montis-alamkuhi* Maassoumi	*-*	*-*	*-*	*Stereothrix*	*Stereothrix*	*Stereothrix*
*A. montismishoudaghi* Sheikh Akbari Mehr, Ghorbani and Maassoumi	*-*	*-*	*-*	*Stereothrix*	*Stereothrix*	*Stereothrix*
*A. montis-varvashti* Podlech	*Stereothrix*	*Stereothrix*	*Stereothrix*	*Stereothrix*	*Stereothrix*	*Stereothrix*
*A. nezva-montis* Podlech and Zarre	*Hypoglottidei*	*Hypoglottidei*	*Hypoglottidei*	*Plagiophaca*	*Plagiophaca*	*Malacothrix*
*A. nurensis* Boiss. and Buhse (*=A. pish-chakensis* Maassoumi)	*Hypoglottidei*	*Hypoglottidei*	*Hypoglottidei*	*Hypoglottidei*	*Hypoglottidei*	*Hypoglottidei*
*A. penetratus* Maassoumi	*Brachylobium*	*Brachylobium*	*Brachylobium*	*Brachylobium*	*Brachylobium*	*Brachylobium*
*A. plagiophacos* Maassoumi and Podlech	*Plagiophaca*	*Plagiophaca*	*Plagiophaca*	*Plagiophaca*	*Plagiophaca*	*Plagiophaca*
*A. podosphaerus* Boiss. and Hausskn.	*Stereothrix*	*Stereothrix*	*Stereothrix*	*Stereothrix*	*Stereothrix*	*Stereothrix*
*A. pseudocapito* Podlech	*Stereothrix*	*Stereothrix*	*Stereothrix*	*Stereothrix*	*Stereothrix*	*Stereothrix*
*A. rimarum* Bornm.	*Hypoglottidei*	*Hypoglottidei*	*Hypoglottidei*	*Hypoglottidei*	*Hypoglottidei*	*Hypoglottidei*
*A. saganlugensis* Trautv.	*Hypoglottidei*	*Hypoglottidei*	*Hypoglottidei*	*Hypoglottidei*	*Hypoglottidei*	*Hypoglottidei*
*A. setosulus* Gontsch.	*-*	*-*	*Stereothrix*	-	*Stereothrix*	*Stereothrix*
*A. sphaeranthus* Boiss.	*Stereothrix*	*Stereothrix*	*Stereothrix*	*Stereothrix*	*Stereothrix*	*Stereothrix*
*A. yazdii* (Vassilcz.) Podlech and Maassoumi	*Brachylobium*	*Brachylobium*	*Brachylobium*	*Brachylobium*	*Brachylobium*	*Brachylobium*

**Table 2:** A summary of the establishment of the relevant sections associated with the *Stereothrix* clade over the years in chronological order.

**Section**	**Established by**	**Year**
*Hypoglottidei*	DC.	1825
*Oroboidei*	A.Gray	1864
*Stereothri*	Bunge	1868
*Malacothrix*	Bunge	1868
*Hemiphaca*	Bunge	1868
*Brachylobium*	Boiss.	1872
*Koelziana*	Širj. and Rech.f.	1953
*Plagiophaca*	Maassoumi and Podlech	1989

**Table 3:** Characteristics of the analyzed datasets.

	ETS	ITS	ETS + ITS	*mat*K	ETS + ITS + *mat*K
Alignment lengths	282	618	900	1153	2053
Constant characteristics	252	459	711	1044	1755
Variable characteristics	30	159	189	109	298
Parsimony-informative characteristics	7	99	106	61	167
MP tree lengths	208	279	318	150	711
Consistency index (CI)	0.78	0.75	0.75	0.76	0.76
Retention index (RI)	0.82	0.86	0.86	0.85	0.86
Sequence evolution model (No. of categories)	F81(1)	SYM + Γ(6)	TrN + I + Γ(6)	TVM + I + Γ(6)	TVM + I + Γ(6)

## Data Availability

The analyzed DNA sequences are available through GenBank.

## Supplementary Materials

The following supporting information can be downloaded at: https://www.mdpi.com/article/10.3390/biology12010138/s1, Table S1: Examined taxa information analyzed in this study including species and section names, locality, and vouchers plus GenBank accession numbers for the sequences. Dataset S1: The file “SI_Stereothrix_matK_ITS_ETS.nex” contains the aligned sequences of *mat*K, ITS, and ETS in Nexus format.
