# Supplementary material for: Dated Phylogeny of Astragalus Section Stereothrix (Fabaceae) and Allied Taxa in the Hypoglottis Clade
Source: Biology (Basel). 2023 Jan 16;12(1):138. doi: 10.3390/biology12010138 (PMC9855906; doi:10.3390/biology12010138)
Supplement: Supplementary file 1 [file biology-12-00138-s001.zip › biology-1888121-supplementary.pdf]

Supplemental online materials

Table S1. Examined taxa information analyzed in this study including species and section names, locality and voucher plus GenBank accession numbers for the sequences.

No.	Taxa	Section	Locality	Voucher	GenBank acc. ITS/ETS/matK
1	*A. aduncus* Willd.	*Onobrychoidei*	Iran	GenBank	KX954888/-/KX955059
2	*A. alaschanus* Bunge ex Maxim.	*Oroboidei*	China	GenBank	KX954889/-/KX955208
3	*A. alopecias* Pall.	*Alopecuroidei*	Iran	GenBank	AB741272.1/JF409729.1/AB741308.1
4	*A. altimontanus* Podlech & Maassoumi	*Stereothrix*	Iran	Babakhanlu & Amin, 15417, TARI, Holotype	OP218887/OP231645/OP231692
5	*A. alyssoides* Lam.	*Hololeuce*	Iran	GenBank	KX954892/-/KX955061
6	*A. andabilensis* Ranjbar & Mahmoudian	*Stereothrix*	Iran	Bidar, s.n., TARI	OP218888/OP231646/OP231693
7	*A. annularis* Forssk.	*Annulares*	Iran	GenBank	KX954893/JF409730.1/KX955062
8	*A. atricapillus* Bornm.	*Hypoglottidei*	Iran	Charkhchian, 2144, TARI	OP218889/OP231647/OP231694
9	*A. australis* (L.) Lam.	*Hemiphragmium*	Unkown	GenBank	KX954898/-/KX955067
10	*A. badelehensis* Maassoumi & Taheri	*Stereothrix*	Iran	Maddah, 2874, TARI, Holotype	OP218890/OP231648/OP231695
11	*A. barbatus* Lam.	*Stereothrix*	Iran	Nydegger, 45613, MSB	OP218891/OP231649/OP231696
12	*A. bavanatensis* Zarre & Podlech	*Stereothrix*	Iran	No collector, 80517, TARI	OP218892/OP231650/OP231697
13	*A. beckii* Bornm.	*Malacothrix*	Iran	GenBank	KX954902/-/KX955071
14	*A. bojnurdensis* Podlech	*Brachylobium*	Iran	Borani & Ramasani, 383, TARI	MN812585/MN812601/OP231698
15	*A. bojnurdensis* Podlech	*Brachylobium*	Iran	Gazeri & Sheikh, 738, TARI, Holotype	MN812586/MN812602/OP231699
16	*A. brachyodontus* Boiss.	*Ornithopodium*	Iran	GenBank	KX954908/-/KX955076
17	*A. brachypetalus* Trautv.	*Stereothrix*	Iran	Termeh, Moussavi & Tehrani, 41381, TARI	OP218893/OP231651/OP231700
18	*A. brachypetalus* Trautv.	*Stereothrix*	Iran	Maassoumi, Pakravan & Nasseh, 72314, TARI	OP218894/OP231652/OP231701
19	*A. brachypetalus* Trautv.	*Stereothrix*	Iran	Akhani, 9390, M	OP218895/OP231653/OP231702
20	*A. brachypetalus* Trautv.	*Stereothrix*	Turkmenistan	Sintenis, 730b, MSB Isotype	OP218896/OP231654/OP231703
21	*A. capito* Boiss. & Hohen.	*Stereothrix*	Iran	Charkhchian, 2129, TARI	OP218897/OP231655/OP231704
22	*A. capito* Boiss. & Hohen.	*Stereothrix*	Iran	Runemark & Maassoumi, 21777, MSB	OP218898/OP231656/OP231705
23	*A. chamberlainianus* H.Sümbül	*Stereothrix*	Turkey	Nydegger, 46384, MSB	OP218899/OP231657/OP231706
24	*A. chamberlainianus* H.Sümbül	*Stereothrix*	Turkey	Ulrich, 9/31, MSB	OP218900/OP231658/OP231707

25	*A. compactus* Lam.	*Rhacophorus*	Iran	GenBank	KX954924/-/KX955091
26	*A. daenensis* Boiss.	*Brachylobium*	Iran	GenBank	KX954930/-/KX955095
27	*A. damghanensis* Podlech	*Stereothrix*	Iran	Wendelbo & Assadi, 29574, TARI, Holotype	OP218901/OP231659/OP231708
28	*A. doshman-ziariensis* Maassoumi & Podlech	*Stereothrix*	Iran	Mozaffarian, 45827, TARI, Holotype	OP218902/OP231660/OP231709
29	*A. doshman-ziariensis* Maassoumi & Podlech	*Stereothrix*	Iran	Zehzad & Taheri, 66872, TARI	OP218903/OP231661/OP231710
30	*A. echinops* Boiss.	*Alopecuroidei*	Iran	GenBank	AB741278.1/JF409737.1/AB741318.1
31	*A. glaucacanthos* Fisch.	*Poterion*	Iran	GenBank	KX954951/-/KX955114
32	*A. griseus* Boiss.	*Malacothrix*	Iran	GenBank	KX954954/-/KX955117
33	*A. hakkariensis* Podlech	*Stereothrix*	Iran	Tarighi & Amini, 1351, TARI	OP218904/OP231662/OP231711
34	*A. hakkariensis* Podlech	*Stereothrix*	Turkey	Nydegger, 18427, MSB, Holotype	OP218905/OP231663/OP231712
35	*A. hakkariensis* Podlech	*Stereothrix*	Turkey	Rechinger, 53839, M	OP218906/OP231664/OP231713
36	*A. hemiphaca* Kar. & Kir.	*Oroboidei*	Kazakhstan	GenBank	KX954959/-/KX955220
37	*A. herbertii* Maassoumi	*Hypoglottidei*	Iran	Terme, Daneshpajuh & Zargani, 39851, TARI, Holotype	MN812587/MN812603/OP231714
38	*A. hymenostegis* Fisch. & C.A.Mey.	*Hymenostegis*	Iran	GenBank	KX954963/-/KX955124
39	*A. inexpectatus* Maassoumi & Podlech	*Malacothrix*	Iran	Nouroozi, 4507, TARI	OP218907/OP231665/OP231715
40	*A. issatissensis* Maassoumi & Mahmoodi	*Irania*	Iran	Mahmoodi & Noroozi, 98654, TARI, Holotype	MW342759/-/MW362137
41	*A. kaufmannii* Krylov	*Hemiphragmium*	Russia	GenBank	KX954970/-/KX955131
42	*A. koelzii* Barneby	*Koelziana*	Iran	Koelz, 15115, W, Isotype	OP218908/OP231666/OP231716
43	*A. ledinghamii* Barneby	*Stereothrix*	Iran	Assadi & Miller, 25179, TARI	OP218909/OP231667/OP231717
44	*A. ledinghamii* Barneby	*Stereothrix*	Iran	Mozaffarian, 44804, TARI	OP218910/OP231668/OP231718
45	*A. ledinghamii* Barneby	*Stereothrix*	Iran	Mozaffarian, 47005, TARI	OP218911/OP231669/OP231719
46	*A. ledinghamii* Barneby	*Stereothrix*	Iran	Mozaffarian, 71268, TARI	OP218912/OP231670/OP231720
47	*A. leucothrix* Freyn & Bornm.	*Stereothrix*	Turkey	Sintenis, 4217, MSB	OP218913/-/OP231721
48	*A. longirostratus* Pau	*Oroboidei*	Iran	No collector, 12345 (s.n), TARI	OP218914/OP231671/OP231722
49	*A. lunatus* Pall	*Ornithopodium*	Iran	GenBank	KX954980/-/KX955141
50	*A. macrourus* Fisch. & C.A.Mey.	*Malacothrix*	Iran	GenBank	KX954983/-/KX955144
51	*A. mahmutlarensis* Podlech	*Stereothrix*	Turkey	Ulrich, A/20, MSB, Holotype	OP218915/OP231672/OP231723

52	*A. mahneshanensis* Maassoumi & Moussavi	*Stereothrix*	Iran	Moussavi, 3957, TARI, Holotype	OP218916/OP231673/OP231724
53	*A. montis-alamkuhi* Maassoumi	*Stereothrix*	Iran	Ghelichkhani, s.n. (4300), TARI, Holotype	OP218917/OP231674/OP231725
54	*A. montis-varvashti* Podlech	*Stereothrix*	Iran	Mozaffarian, 81162, TARI	OP218918/OP231676/OP231727
55	*A. montismishoudaghi* Sheikh Akbari Mehr, Ghorbani & Maassoumi	*Stereothrix*	Iran	Sheikh-Akbari Mehr & Ghorbani, 92499, TARI, Holotype	OP218919/OP231675/OP231726
56	*A. nezva-montis* Podlech & Zarre	*Malacothrix*	Iran	Wendelbo, 1282, W, Holotype	OP218920/OP231677/OP231728
57	*A. nezva-montis* Podlech & Zarre	*Malacothrix*	Iran	Maddah, 2410, TARI	OP218921/OP231678/OP231729
58	*A. nurensis* Boiss. & Buhse	*Stereothrix*	Iran	Assadi & Maassoumi, 51549, TARI	MN812588/MN812604/OP231730
59	*A. penetratus* Maassoumi	*Brachylobium*	Iran	GenBank	KX955003/-/KX955162
60	*A. perpexus* Maassoumi (=*A. longirostratus*)	*Oroboidei*	Iran	Nouroozi & Rahiminejad, 1602, TARI	OP218922/OP231679/OP231731
61	*A. pish-chakensis* Maassoumi (=*A. nurensis*)	*Hypoglottidei*	Iran	Assadi & Maassoumi, 51407, TARI	MN812592/MN812608/OP231732
62	*A. pish-chakensis* Maassoumi (=*A. nurensis*)	*Hypoglottidei*	Iran	Maassoumi, 75471, TARI	MN812591/MN812607/OP231733
63	*A. plagiophacos* Maassoumi & Podlech	*Plagiophaca*	Iran	GenBank	AB231132.1/-/-
64	*A. podosphaerus* Boiss. & Hausskn.	*Stereothrix*	Iran	Kalvandi & Faramarzi, 2085, TARI	OP218923/OP231680/OP231734
65	*A. podosphaerus* Boiss. & Hausskn.	*Stereothrix*	Iran	Ranjbar, 2590, TARI	OP218924/OP231681/OP231735
66	*A. podosphaerus* Boiss. & Hausskn.	*Stereothrix*	Iran	Lamond & Termeh, 42598, MSB	OP218925/OP231682/OP231736
67	*A. pseudocapito* Podlech	*Stereothrix*	Iran	Termeh & Daneshpajouh, 41369, MSB, Holotype	OP218926/OP231683/OP231737
68	*A. pseudocapito* Podlech	*Stereothrix*	Iran	Mozaffarian & Ramezani, 96404, TARI	OP218927/OP231684/OP231738
69	*A. rimarum* Bornm.	*Hypoglottidei*	Iran	Maassoumi, 59427, TARI	OP218928/OP231685/OP231739
70	*A. saganlugensis* Trautv.	*Hypoglottidei*	Iran	Maassoumi & Bagheri, 98378, TARI	MN812598/MN812614/OP231740
71	*A. saganlugensis* Trautv.	*Hypoglottidei*	Turkey	Meierott & Gergor, s.n. 2010, MSB	OP218929/OP231686/OP231741
72	*A. saganlugensis* Trautv.	*Hypoglottidei*	Armenia	Akhverdov, 0076367, W	OP218930/OP231687/OP231742
73	*A. saganlugensis* Trautv.	*Hypoglottidei*	Iran	Szovits, 1889-0152923, W	OP218931/OP231688/OP231744
74	*A. saganlugensis* Trautv.	*Hypoglottidei*	Armenia	Akhverdov, 2006-0007466, W	OP218932/OP231689/OP231743

75	*A. setosulus* Gontsch.	*Stereothrix*	Ukraine	Krijukova, s.n. (1963), MSB	OP218933/OP231690/OP231745
76	*A. sphaeranthus* Boiss.	*Stereothrix*	Iran	Riazi, 10230, TARI	MN812599/MN812615/OP231746
77	*A. sphaeranthus* Boiss.	*Stereothrix*	Iraq	Rechinger, 11434, MSB	OP218934/OP231691/OP231747
78	*A. sphaeranthus* Boiss.	*Stereothrix*	Iran	Mozaffarian, 96975, TARI	MN812600/MN812616/OP231748
79	*A. vegetus* Bunge	*Onobrychoidei*	Iran	GenBank	KX955046/-/KX955203
80	*A. yazdii* (Vassilcz.) Podlech & Maassoumi	*Brachylobium*	Iran	GenBank	KX954886/-/KX955057
81	*Colutea persica* Boiss.	-	Iran	GenBank	KX954877/-/KX955051
82	*Oxytropis aucheri* Boiss.	-	Iran	GenBank	LC213309.1/JF409764.1/KM387602.1
83	*Oxytropis pilosa* (L.) DC.		Armenia	GenBank	KX954880/-/KX955052
